# Autoregulation of ToxR and Its Regulatory Actions on Major Virulence Gene Loci in *Vibrio parahaemolyticus*

**DOI:** 10.3389/fcimb.2018.00291

**Published:** 2018-09-05

**Authors:** Yiquan Zhang, Lingfei Hu, George Osei-Adjei, Ying Zhang, Wenhui Yang, Zhe Yin, Renyun Lu, Xiumei Sheng, Ruifu Yang, Xinxiang Huang, Dongsheng Zhou

**Affiliations:** ^1^School of Medicine, Jiangsu University, Zhenjiang, China; ^2^State Key Laboratory of Pathogen and Biosecurity, Beijing Institute of Microbiology and Epidemiology, Beijing, China

**Keywords:** *Vibrio parahaemolyticus*, ToxR, T3SS1, Vp-PAI, virulence

## Abstract

*Vibrio parahaemolyticus*, the leading causative agent of seafood-associated gastroenteritis, harbors two major virulence gene loci T3SS1 and Vp-PAI (T3SS2 and *tdh2*). ToxR is a virulence regulator of vibrios. Cell density-dependent transcriptional pattern of *toxR* and its regulatory actions on T3SS1 and Vp-PAI have been previously reported, but the detailed regulatory mechanisms are still obscure. In the present work, we showed that the highest transcription level of *toxR* occurs at an OD_600_ = 0.2–0.4, which may be due to the subtle repression of ToxR and the quorum-sensing (QS) master regulator AphA. We also showed that ToxR is involved in regulating the mouse lethality, enterotoxicity, cytotoxicity, and hemolytic activity of *V. parahaemolyticus*. ToxR binds to the multiple promoter-proximal DNA regions within the T3SS1 locus to repress their transcription. In addition, ToxR occupies the multiple promoter-proximal DNA regions of Vp-PAI locus to activate their transcription. Thus, ToxR regulates the multiple virulence phenotypes via directly acting on the T3SS1 and Vp-PAI genes. Data presented here provide a deeper understanding of the regulatory patterns of ToxR in *V. parahaemolyticus*.

## Introduction

*Vibrio parahaemolyticus* is a Gram-negative halophilic bacterium that is highly abundant in estuarine and marine environments (Thompson et al., 2004). It is a well-known seafood-borne human pathogen that can cause acute gastroenteritis via consumption of raw or poorly cooked, contaminated seafood (Thompson et al., 2004). The major clinical symptoms include chills, fever, nausea, vomiting, watery diarrhea, and abdominal cramps (Broberg et al., 2011). Several large-scale outbreaks of *V. parahaemolyticus* infections have occurred since 1996, which are associated with the O3:K6 serovar and its serovariants (Yeung and Boor, 2004; Nair et al., 2007). Strains of the serotype O3:K6 and its genetic variants were known as the pandemic group of *V. parahaemolyticus* with higher levels of virulence relative to other groups (Yeung and Boor, 2004; Nair et al., 2007).

The pandemic *V. parahaemolyticus* strain RIMD2210633 expresses multiple virulence determinants including major ones, such as thermostable direct hemolysin (TDH) and type III secretion system 1 (T3SS1) and system 2 (T3SS2) (Makino et al., 2003). The TDH can cause β-type hemolysis when the bacterium is grown on the special Wagatsuma agar, a phenomenon known as the Kanagawa phenomenon (KP) (Miyamoto et al., 1969; Honda et al., 1988). The TDH is also required for the lethality in mice and partially contributes to the cytotoxicity and enterotoxicity of *V. parahaemolyticus* (Raimondi et al., 2000; Naim et al., 2001; Park et al., 2004a; Hiyoshi et al., 2010). The strain harbors two copies of *tdh* (*tdh2*:VPA1314 and *tdh1*:VPA1378) on the smaller chromosome II (Makino et al., 2003). However, the production of *tdh2* is predominantly responsible for the TDH activities because of much higher transcriptional levels of *tdh2* than that of *tdh1* (Nishibuchi and Kaper, 1990; Okuda and Nishibuchi, 1998). The two copies of *tdh* genes together with T3SS2 (VPA1320-1370) locus are located on the 80 kb pathogenicity island termed Vp-PAI (VPA1312-1398) (Makino et al., 2003). The T3SS1 mainly contributes to the cytotoxicity of *V. parahaemolyticus* that induces a series of events including autophagy, membrane blebbing, and, lastly, cell lysis (Park et al., 2004b; Hiyoshi et al., 2010; Letchumanan et al., 2014). By contrast, T3SS2 has been demonstrated to be involved in enterotoxicity of *V. parahaemolyticus* that causes the accumulation of intestinal fluid in a rabbit ileal loop model (Park et al., 2004b; Hiyoshi et al., 2010). The genetic organizations of both T3SS1 (VP1656-VP1702) and T3SS2 are complex gene clusters that are composed of more than 40 consecutive genes, respectively (Makino et al., 2003).

The ToxR is a membrane-localized regulatory protein that plays an essential role in modulating bacterial persistence and virulence (Crawford et al., 2003; Childers and Klose, 2007). *V. cholerae* ToxR binds to the promoter-proximal DNA region of *toxT* to enhance its transcription, and ToxT, in turn, activates the virulence genes including *ctx* and *tcp* encoding toxin coregulated pilus (TCP) and cholera toxin (CT), respectively (Dirita et al., 1991; Higgins and Dirita, 1994). The ToxR also can directly activate *ctx* transcription in a ToxT-independent manner in the presence of bile acids (Hung and Mekalanos, 2005). Additionally, some outer membrane proteins (Omps), such as *ompU* and *ompT*, as well as biofilm formation are also under the control of ToxR, suggesting that ToxR is necessary for the survival of the bacteria under adverse environmental stimuli (Provenzano and Klose, 2000; Provenzano et al., 2001; Valeru et al., 2012). *V. parahaemolyticus* ToxR is highly similar to *V. cholerae* ToxR (Lin et al., 1993). It has been demonstrated that ToxR is involved in regulating the expression of TDH, T3SS1 as well as T3SS2 genes in *V. parahaemolyticus* (Lin et al., 1993; Whitaker et al., 2012; Hubbard et al., 2016). A previous study showed that ToxR represses the transcription of T3SS1 genes most likely via direct activation of CalR, which is a direct repressor of T3SS1 (Osei-Adjei et al., 2017). However, whether ToxR has direct regulatory effects on T3SS1 genes and the detailed regulatory actions of ToxR on *tdh* and T3SS2 genes are still obscure. In addition, cell density-dependent transcriptional patterns of *toxR* have been observed in *V. cholerae* and *V. parahaemolyticus* (Xu et al., 2010; Zhang et al., 2017), which suggest a possible connection between ToxR expression and quorum sensing (QS) in pathogenic vibrios. However, the molecular mechanisms also need to be further investigated.

The QS is a cell–cell signaling process that bacteria use to modulate communal behavior and gene regulation in response to cell density changes and the presence of chemical molecules termed autoinducers (AIs) in the surroundings (Srivastava and Waters, 2012). The QS controls gene expression generally via the downstream master regulators (Ng and Bassler, 2009). The AphA and OpaR represent the two master regulators of QS in *V. parahaemolyticus* that function at low cell density (LCD) and high cell density (HCD), respectively (Sun et al., 2012; Zhang et al., 2012). In the present work, we showed that AphA indirectly represses the transcription of *toxR* at LCD, whereas OpaR has no regulatory actions on *toxR* transcription. Meanwhile, ToxR also shows no regulatory actions on both *aphA* and *opaR*. However, autorepression of ToxR was observed herein. In addition, ToxR acts as a virulence regulator of *V. parahaemolyticus* via direct repression of T3SS1 genes, but activates Vp-PAI (T3SS2 and *tdh2*) genes. This work promotes us to gain a deeper understanding of the regulatory patterns of ToxR in *V. parahaemolyticus*.

## Materials and methods

### Bacterial strains and growth conditions

*V. parahaemolyticus* RIMD2210633 was used as the wild-type (WT) strain in the present work (Makino et al., 2003). The nonpolar *aphA, opaR*, and *toxR* single-gene deletion mutants derived from WT (designated as Δ*aphA*, Δ*opaR*, and Δ*toxR*, respectively), and the corresponding complementary mutants were previously described (Sun et al., 2012; Zhang et al., 2012, 2017). All the primers used are listed in Table 1.

**Table 1:** Oligonucleotide primers used in this study.

**Target**	**Primers (forward/reverse, 5′-3′)**
**CONSTRUCTION OF MUTANTS**	
*opaR*	GTGACTGCAGACTGCCTTGGTAACGCTCTG /GTTCGTGTTCAAATCTGAGCTATCCATTTTCCTTGCCATTTG
	CAAATGGCAAGGAAAATGGATAGCTCAGATTTGAACACGAAC /GTGAGCATGCATGGGCTGCATCAGGTCG
	GTGACTGCAGACTGCCTTGGTAACGCTCTG /GTGAGCATGCATGGGCTGCATCAGGTCG
*aphA*	GTGACTGCAGCGCAGCAAATAACCAGAC /CCAATCACTTCAAGTTCTGTTGTCTTCAATCCAAATGGTC
	GACCATTTGGATTGAAGACAACAGAACTTGAAGTGATTGG /GTGAGCATGCGTTTTCGTGACCGCTGTG
	GTGACTGCAGCGCAGCAAATAACCAGAC /GTGAGCATGCGTTTTCGTGACCGCTGTG
*toxR*	GTGACTGCAGAAACGCAATTTGTCTGATG /ATCTTCATGCTGGCCTCCTTTAGTTCTTCTTAGATGGATGATG
	CATCATCCATCTAAGAAGAACTAAAGGAGGCCAGCATGAAGAT /GTGAGCATGCAATTCGGCGGCTTTGTTC
	GTGACTGCAGAAACGCAATTTGTCTGATG /GTGAGCATGCAATTCGGCGGCTTTGTTC
**CONSTRUCTION OF COMPLEMENTED MUTANTS**	
*toxR*	GATTCTAGAAGGAGGAATTCACCATGACTAACATCGGCACCAA /GACAAGCTTTTATTTGCAGATGTCTGTTGG
**PROTEIN EXPRESSION**	
*opaR*	AGCGGGATCCATGGACTCAATTGCAAAGAG /AGCGAAGCTTTTAGTGTTCGCGATTGTAG
*aphA*	AGCGGGATCCATGTCATTACCACACGTAATC /AGCGAAGCTTTTAACCAATCACTTCAAGTTC
*toxR*	AGCGGGATCCATGACTAACATCGGCACCAA /AGCGAAGCTTTTAAGGATTCACAGCAGAAG
**qPCR**
*aphA*	AGCATCGGTTACTTCTGGAAAG/GTTGAACAGCACAAGCCATAAG
*opaR*	TGTCTACCAACCGCACTAACC/GCTCTTTCAACTCGGCTTCAC
*toxR*	TTGTTTGGCGTGAGCAAGG/TAGCAGAGGCGTCATTGTTATC
*exsB*	ATGAAAAGCAGTAAGTGGGC/CTGAGAAGCAACAGTAAGAC
VP1687	TGCTCACCGTTGCCAAATAG/GCGACGCTTTCATGTATTGC
*vopN*	GGAATGGATTGGAATCGTC/CCACCGTCTTTTATTTTGC
*vtrA*	AGTCTAGGCTCACAAGATCG/AAATGGGCTCTGATGTTACG
*vopB2*	ACCAGCCTCAGCAACAAGC/CTTTCACGAATACTACGC
*tdh2*	ATGTAAAAAGAAAACCGTACA/AACACAGCAGAATGACCGTG
**PRIMER EXTENSION**	
*aphA*	/GCTCTTACTGGCGCTTGAG
*opaR*	/ATCCATTTTCCTTGCCATTTG
*toxR*	/TTAGTTCTTCTTAGATGGATGATG
*exsB*	/GTCTTATTATGATTTATTTTTACAC
VP1687	/GGCAACGGTGAGCAAAATC
*vopN*	/GACGATTCCAATCCATTCCG
*vtrA*	/CCGCTATCGCTGCTATTT
*vopB2*	/GAGATTCGTAGCGTATAAGTGC
*tdh2*	/GCAAAATATCGGTACTTCA
**LacZ FUSION**	
*aphA*	GCGCGTCGACCATTCGTAATACAAAAGG /GCGCGGTACCTTCCAGAAGTAACCGATGCTAG
*opaR*	GCGCGTCGACTCCATCGTGTTGCCGTAGC /GCGCGGTACCCAATATCTGCGTGACCACCAC
*toxR*	GCGCGTCGACATCGTTAAGGTATTTGCA /GCGCGAATTCCGAGCGAATTACTATTTGG
*exsB*	ATATGTCGACATTGTCCGTCAAATGCAGTTC /TTTTGAATTCCATATACATTCGCTTGGCTCTG
VP1687	GCGCGTCGACGCATTATTGACGCCAGTATCG /GCGCTCTAGAGGCAACGGTGAGCAAAATC
*vopN*	GCGGTCGACCAGATTGCTGAATATCGGTG /GCGTCTAGAAAGCGATTGAGTGGCGTTG
*vtrA*	GCGCGTCGACTACGCTTCCAATAATCACC /GCGCGAATTCCCGATCTTGTGAGCCTAGA
*vopB2*	GCGGTCGACGCGTACTAAGTGATGAAGAG /GCGTCTAGACAACAGAACCACTTTCAGC
*tdh2*	GCGCGTCGACAATTCACGACGAATCGGAG /GCGCGAATTCATATCGGTACTTCATAAA
**EMSA**	
*aphA*	CATTCGTAATACAAAAGG/TTCCAGAAGTAACCGATGCTAG
*opaR*	TCCATCGTGTTGCCGTAGC/CAATATCTGCGTGACCACCAC
*toxR*	ATCGTTAAGGTATTTGCA/CGAGCGAATTACTATTTGG
*exsB*	ATTGTCCGTCAAATGCAGTTC/CATATACATTCGCTTGGCTCTG
VP1687	GCATTATTGACGCCAGTATCG/GGCAACGGTGAGCAAAATC
*vopN*	CAGATTGCTGAATATCGGTG/AAGCGATTGAGTGGCGTTG
*vtrA*	TACGCTTCCAATAATCACC/CCGATCTTGTGAGCCTAGA
*vopB2*	GCGTACTAAGTGATGAAGAG/CAACAGAACCACTTTCAGC
*tdh2*	AATTCACGACGAATCGGAG/ATATCGGTACTTCATAAA
**DNase I FOOTPRINTING**	
*toxR*	TTTCAGGGACGACTTTGTG/TTAGTTCTTCTTAGATGGATGATG
VP1687	CACCAGAGTAGGGCATCAC/CAGAGTGACCCAGAGCCG
*vopN*	CAGATTGCTGAATATCGGTG/ATTGATAATACTCATTCACTTGC
*vtrA*	CATTGCCCAAGTTTATCAG/CCGATCTTGTGAGCCTAGAC
*tdh2*	TCATTACCACAACGCCTCTG/CTGTGATTCCTCAAGCGG

The 2.5% Bacto heart infusion (HI) broth (BD Bioscience, USA) was used to culture *V. parahaemolyticus* strains. Overnight bacterial cell cultures were diluted 1:50 into 15 ml of fresh HI broth, and allowed to grow at 37°C with shaking at 200 rpm to reach OD_600_≈1.0 (the midexponential growth phase), and then diluted 1:1000 into 15 ml of fresh HI broth and allowed to grow under the same conditions to reach the required cell densities. When necessary, the medium was supplemented with 50 μg/ml gentamicin, 5 μg/ml chloramphenicol, or 0.1% arabinose.

### Murine infection assay

The murine infection assay was done as previously described (Sun et al., 2014). Briefly, 0.1 ml of the 10^8^ CFU/ml bacterial suspensions in phosphate buffered saline (PBS) (pH7.2) was inoculated intraperitoneally into each of the 15 female BALB/c mice that were 25 to 28 days old, after which the number of mice killed at specified times was monitored daily. The PBS was used as a control in one trial to confirm experimental outcomes.

### Kanagawa phenomenon (KP) test

The KP test was done as previously described (Honda et al., 1980). Briefly, 5 μl of the third-round cell cultures were inoculated onto Wagatsuma agar medium containing 5% rabbit red blood cells (RBCs), 5 μg/ml chloramphenicol, and 0.1% arabinose. The radius from the point of inoculation to the edge of the β-hemolysin zone was measured after static incubation at 37°C for at least 24 h.

### Cytotoxicity assay

The cytotoxicity assay was performed as previously described (Hiyoshi et al., 2010; Sun et al., 2014). Briefly, the third-round cell cultures were washed and serially diluted with the prewarmed Dulbecco's modified Eagle's medium (DMEM) lacking phenol red for CFU measurement and infection. HeLa cells were infected with 10^6^ CFU of bacteria for 3 h at a multiplicity of infection (MOI) of 2.5. Subsequently, the release of lactate dehydrogenase (LDH) into the medium was quantified with a CytoTox 96® Nonradioactive Cytotoxicity Assay kit (Promega, USA) according to the manufacturer's instructions.

### Rabbit ileal loop test

The rabbit ileal loop test was done as previously described (Nishibuchi et al., 1992; Sun et al., 2014). Briefly, four loops (10 cm each in length) were placed in the small intestine of each of the six rabbits. One milliliter of bacterial suspension (10^9^ CFU/ml) was injected into each ileal loop. The rabbits were sacrificed by the venous air embolism 14 h postinjection, and the fluid accumulation was calculated as the amount of accumulated fluid of each ligated rabbit ileal loop. Isoflurane was employed for the inhalation anesthesia of rabbits before each surgery. All the animal experiments were approved by the Committee on Animal Research of the Academy of Military Medical Sciences and carried out per the approved guideline.

### Quantitative PCR (qPCR)

Total RNAs were extracted from *V. parahaemolyticus* strains using the TRIzol Reagent (Invitrogen, USA). The contaminating genomic DNA in the total RNAs was removed using the Ambion's DNA-free™ Kit according to the manufacturer's instructions. The cDNAs were generated by using 8 μg of total RNAs and 3 μg of random hexamer primers. The SYBR Green qPCR assay was performed and analyzed as previously described (Gao et al., 2011). The relative mRNA levels of each target gene were determined based on the standard curve of 16S rRNA (reference gene) expression for each RNA preparation.

### Primer extension assay

The primer extension assay was done as previously described (Gao et al., 2011; Zhang et al., 2012). Briefly, about 10 μg of total RNAs were annealed with 1 pmol of 5′- ^32^P-end labeled reverse oligonucleotide primer to generate cDNAs using a Primer Extension System (Promega, USA). The same labeled primer was used for sequencing with the AccuPower and Top DNA Sequencing Kit (Bioneer, Korea). The primer extension products and sequencing materials were concentrated and analyzed in an 8M urea−6% polyacrylamide gel electrophoresis, and the results were detected by autoradiography with the Fuji Medical X-ray film (Fuji Photo Film Co., Ltd. Japan).

### LacZ fusion and β-galactosidase assay

For the LacZ fusion assay (Gao et al., 2011; Sun et al., 2012), the regulatory DNA region of each indicated gene was cloned into the corresponding restriction endonuclease sites of the pHRP309 plasmid harboring a promoterless *lacZ* reporter gene and a gentamicin-resistance gene (Parales and Harwood, 1993). The resultant plasmid was then transferred into *V. parahaemolyticus* strains, in which the promoterless *lacZ* gene can be expressed under the control of the target promoters. Thus, the regulatory actions of ToxR on target genes can be assessed by measuring the β-galactosidase activities in cellular extracts of WT and Δ*toxR* (β-Galactosidase Enzyme Assay System, Promega).

### Preparation of 6 × His-tagged proteins

The entire coding regions of *aphA, opaR*, and the truncated *toxR* (1-528 bp, a.a.1-176) were cloned into plasmid pET28a (Novagen, USA), respectively. The recombinant plasmids encoding His-tagged proteins were transferred into *E. coli* BL21λDE3 cells for protein expression (Kleber-Janke and Becker, 2000). The conditions for expression and purification of His-tagged AphA, OpaR, and ToxR have been described previously (Sun et al., 2012; Zhang et al., 2012, 2017).

### Electrophoretic mobility shift assay (EMSA)

The EMSA was done as previously described (Sun et al., 2012; Zhang et al., 2012, 2017). Briefly, the 5′-ends of the promoter-proximal DNA region of each target gene were labeled using [γ-^32^P] ATP and T4 polynucleotide kinase. DNA binding was performed in a 10 μl reaction volume containing binding buffer (1 mM MgCl_2_, 0.5 mM EDTA, 0.5 mM DTT, 50 mM NaCl, 10 mM Tris-HCl/pH 7.5, and 10 mg/ml salmon sperm DNA), labeled DNA probe (about 2,000 CPM/μl), and increasing amounts of His-tagged protein. After being incubated at room temperature for 30 min, the products were loaded onto a native 4% (w/v) polyacrylamide gel, and electrophoresed in 0.5 × TBE buffer for about 50 min at 200 V. Radioactive species were detected by autoradiography after exposure to Fuji Medical X-ray film at −20°C. Three controls were included in each EMSA experiment: (1) cold probe as specific DNA competitor (the same promoter-proximal DNA region unlabeled), (2) negative probe as nonspecific DNA competitor (the unlabeled coding region of the 16S rRNA gene), and (3) nonspecific protein competitor (rabbit anti-F1-protein polyclonal antibodies).

### DNase I footprinting

The DNase I footprinting was done as previously described (Sun et al., 2012; Zhang et al., 2012). Briefly, single strand ^32^P-5′ end-labeled probes were incubated with increasing amounts of His-tagged protein for 30 min at room temperature, in a final 10 μl reaction volume containing the binding buffer used in EMSA. Before DNA digestion, 10 μl of Ca^2+^/Mg^2+^ solution (5 mM CaCl_2_ and 10 mM MgCl_2_) was added, followed by incubation for 1 min at room temperature. The optimized RQ1 RNase-Free DNase I (Promega, USA) was then added to the reaction mixture and the mixture was incubated at room temperature for 40 to 90 s. The reaction was quenched by adding 9 μl of stop solution (200 mM NaCl, 30 mM EDTA, and 1% SDS), followed by incubation for 1 min at room temperature. The partially digested DNA samples were extracted with phenol/chloroform, precipitated with ethanol, and analyzed in 6% polyacrylamide/8 M urea gel. Protected regions were identified by comparison with the sequence ladders. The templates for DNA sequencing were the same as the DNA fragments for DNase I footprinting assay. Radioactive species were detected by autoradiography after exposure to Fuji Medical X-ray film at −20°C.

### Prediction of the minimal ToxR binding sites within target regulatory regions

The 500 bp upstream DNA regions of the genes tested (Table 2) were retrieved from the genome sequence of RIMD 2210633 with the “*retrieve-seq*” program (Van Helden, 2003). Subsequently, the DNA binding box of ToxR (Goss et al., 2013) was used to statistically predict the presence of ToxR box-like sequences within the target upstream DNA regions by using the *matrices-paster* tool (Van Helden, 2003). This analysis generated the weight scores for each target upstream DNA region. The higher score values represented the higher probability of ToxR and the upstream DNA region association.

**Table 2:** Predicted ToxR box-like sequences within upstream DNA regions.

**Operon**	**First gene**		**ToxR box-like sequence**		
	**ID**	**Name**	**Position^&^**	**Sequene**	**Score**
*toxRS*	VP0820	*toxR*	D-99…-85	TAAAAGCATCTAAAA	10.1
	VP2762	*aphA*	D-333…-319	AAAAAACCCATAAAA	7.6
	VP2516	*opaR*	NA	NA	NA
					
*exsBAD-vscBCD*	VP1700	*exsB*	NA	NA	NA
VP1687-1686	VP1687		D-156…-142	TCAAACGTCTTAAAA	8.5
					
VPA1332-1333	VPA1332	*vtrA*	NA	NA	NA
VPA1362-1358	VPA1362	*vopB2*	NA	NA	NA
	VPA1314	*tdh2*	D-326…-312	TAAAATGACTTAAAT	10.4
			D-316…-302	TAAATCAAAATAAAA	8.5

&, “D” indicates the direct sequence while “R” the reverse one; minus numbers denote the nucleotide positions upstream of indicated genes; “NA” represents “not applicable.”

### Experimental replicates and statistical methods

The LacZ fusion and qPCR assays were performed with at least three independent bacterial cultures, and the values were expressed as the mean ± standard deviation (SD). Paired Student's *t-*test was used to calculate statistically significant differences, *p* < 0.01 was considered to indicate statistical significance. The data for phenotype, primer extension, EMSA, and DNase I footprinting assays were done at least two independent times.

## Results

### Cell density-dependent transcription and autorepression of ToxR

**Figure 1:**
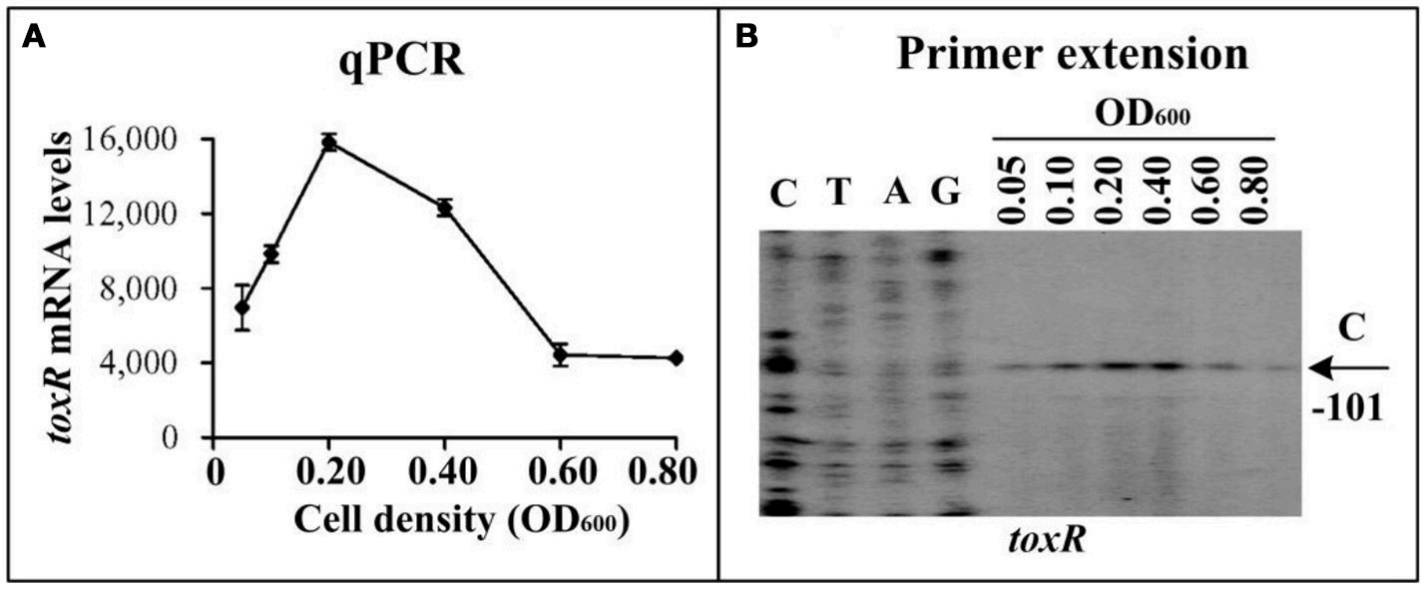
Cell density-dependent transcription of *toxR*. The WT strain was grown in HI broth at 37°C, and bacterial cells were harvested at various OD_600_ values. **(A)** qPCR. The relative mRNA level of each target gene was tested in WT at different cell densities. **(B)** primer extension. An oligonucleotide primer was designed to be complementary to the RNA transcript of *toxR*. The primer extension products were analyzed with an 8 M urea-6% acrylamide sequencing gel. Lanes C, T, A, and G represented Sanger sequencing reactions. The transcriptional start sites were indicated by arrows with nucleotides and positions. The minus numbers under the arrows indicated the nucleotide positions upstream of start codon of *toxR*.

The qPCR and primer extension assays were employed to measure the transcription change of *toxR* at the different growth phases of WT in HI broth (Figure 1). The results showed that the mRNA level of *toxR* increased considerably with the increase of cell density from an OD_600_ value of 0.05 to 0.2, and the highest level occurred at an OD_600_ value of 0.2 to 0.4, but it dramatically decreased when the OD_600_ value was higher than 0.4 (Figure 1). These results suggested that transcription of *toxR* is very likely to be regulated by QS in *V. parahaemolyticus*. AphA and OpaR are the two bottom regulators of QS in *V. parahaemolyticus* operating at LCD and HCD, respectively (Sun et al., 2012; Zhang et al., 2012; Lu et al., 2018). Thus, bacterial cells were harvested at an OD_600_ value of 0.15 and 0.8, respectively, to investigate the regulatory actions of AphA and OpaR on *toxR* transcription. The qPCR and primer extension results showed that the mRNA level of *toxR* increased in Δ*aphA* relative to that in WT (Supplementary Figures 1A,B). The LacZ fusion assay further indicated that the promoter activity of *toxR* was much higher in Δ*aphA* than that in WT (Supplementary Figure 1C). The EMSA result showed that His-AphA was unable to bind to the upstream DNA fragment of *toxR* in a dose-dependent manner (Supplementary Figure 1D). However, His-AphA was able to bind in a dose-dependent manner to the upstream DNA regions of other targets such as *aphA, qrr4*, and *opaR* even at a much lower protein amount (Sun et al., 2012; Zhou et al., 2013). Thus, AphA indirectly repressed *toxR* transcription at LCD. By contrast, OpaR seems to have no regulatory action on *toxR* at HCD (Supplementary Figure 2). These results cannot explain the transcriptional patterns of *toxR* observed in Figure 1, so there must be an additional unknown regulator (s) that regulate *toxR*.

**Figure 2:**
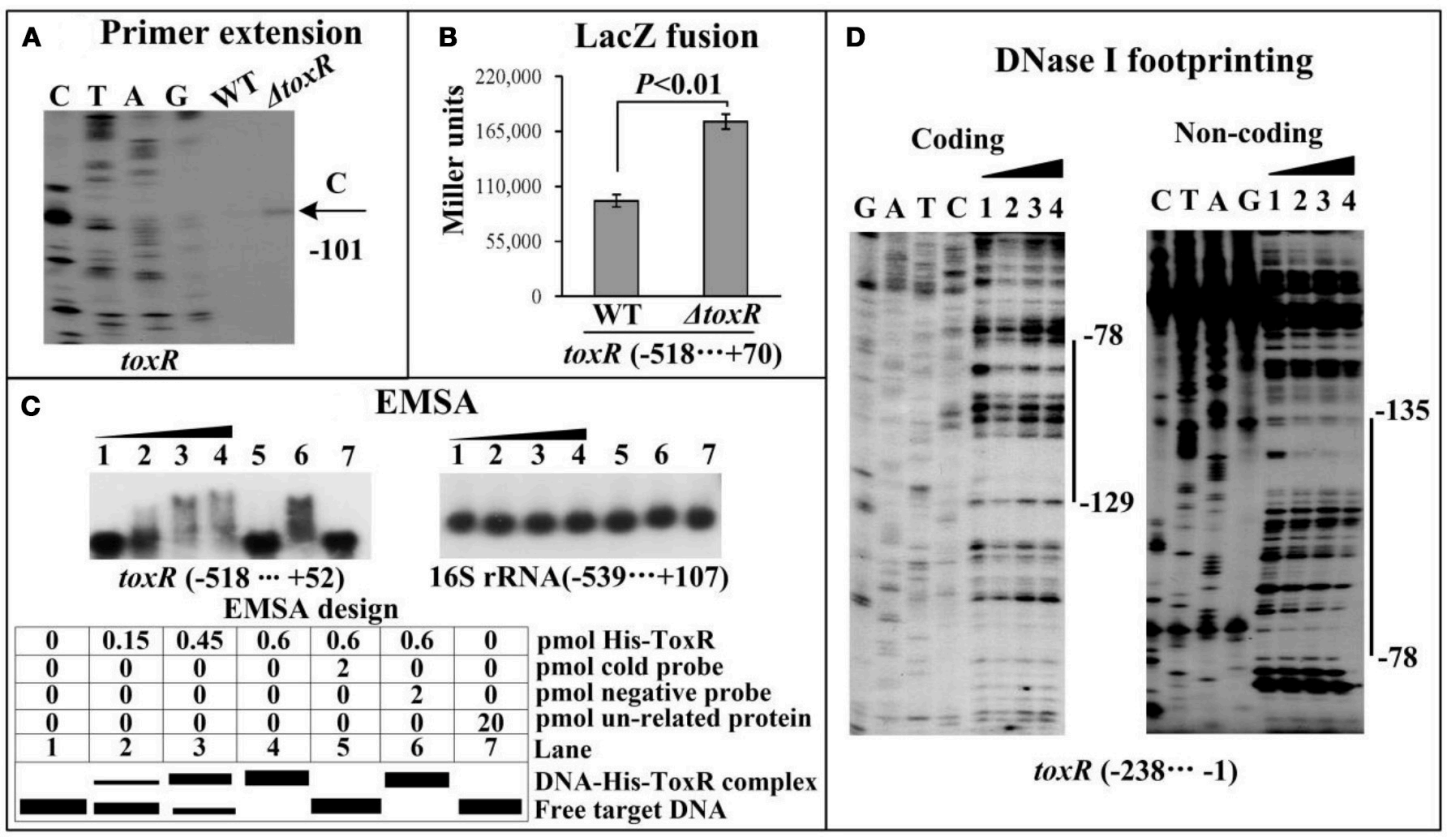
Autoregulation of ToxR. The primer extension **(A)** was done as Figure 1, while the LacZ fusion **(B)** and EMSA **(C)** were done as Supplementary Figure 1. **(D) DNase I footprinting**. Labeled coding or noncoding DNA probes were incubated with increasing amounts of purified His-ToxR proteins, and then subjected to DNase I footprinting assay. The footprint regions were indicated by vertical bars with positions.

A ToxR box-like sequence (TAAAAGCATCTAAAA, also shown in Table 2) was detected within the promoter-proximal DNA region of *toxR* by using the DNA binding box of ToxR and the online *matrix-scan* tool (http://embnet.ccg.unam.mx/rsat/), suggesting an autoregulation mechanism of ToxR in *V. parahaemolyticus*. Thus, the bacterial cells were harvested at an OD_600_ value of 0.4 to investigate the autoregulation of ToxR. Since the *toxR* coding region was deleted from the *V. parahaemolyticus* genome, we thus chose a reverse primer located upstream of *toxR* but next to the start codon to conduct the primer extension assay (Table 1). As shown in Figure 2A, a single transcription start site located at 101 bp upstream of *toxR* was detected, and its transcriptional activity was hugely enhanced in Δ*toxR* relative to WT. The recombinant *lacZ* fusion plasmid that contains the regulatory region of *toxR* and a promoterless *lacZ* gene was transferred into Δ*toxR* and WT, respectively, to test the action of ToxR on its own promoter. The result showed a significantly enhanced promoter activity of *toxR* in Δ*toxR* relative to WT (Figure 2B). *In vitro* EMSA results showed that His-ToxR could bind to its own regulatory region in a dose-dependent manner, but it could not bind to the 16S rRNA gene as the negative control (Figure 2C). As further determined by DNase I footprinting assay (Figure 2D), His-ToxR protected a single DNA region upstream of *toxR* against DNase I digestion, which was considered as the ToxR site. Thus, ToxR represses its own gene transcription in a direct manner.

We further investigated the ToxR-mediated *aphA* and *opaR* transcription. The qPCR, primer extension, and LacZ fusion assays showed that ToxR does not regulate the transcription of both *aphA* and *opaR* (Supplementary Figure 3). The EMSA results showed that His-ToxR was unable to specifically bind to the upstream DNA fragments of *aphA* and *opaR* in a dose-dependent manner (Supplementary Figure 3).

Taken together, AphA indirectly represses *toxR* at LCD and autorepression of ToxR at the transition from LCD to HCD (OD_600_ = 0.2–0.4) may result in the cell-density transcription pattern of *toxR*.

### Involvement of ToxR in virulence

**Figure 3:**
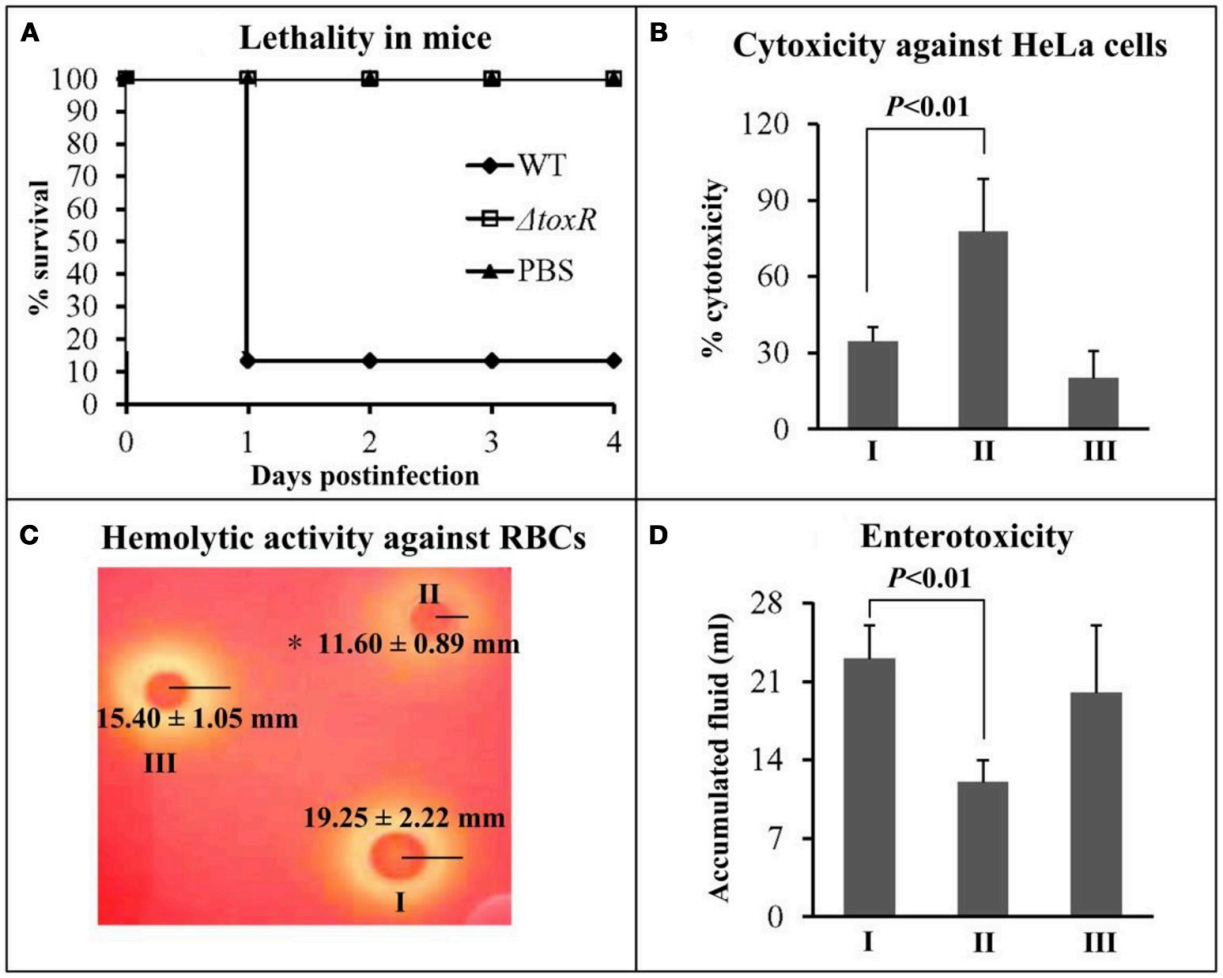
Regulation of ToxR on virulence. I, II, and III represent WT/pBAD33, Δ*toxR*/pBAD33, and C-Δ*toxR*, respectively. **(A)** The mice survival rates infected with *V. parahaemolyticus* strains were measured to determine the lethality in mice. **(B)** The cytotoxicity against HeLa cells was evaluated in terms of the release of LDH. **(C)** The hemolytic activity against RBCs was evaluated by measuring the radius of the β-hemolysin zone. Shown is a representative image of *V. parahaemolyticus* cells on Wagatsuma agar. Values are the mean±SD from three independent experiments with at least four replicates. The symbol * represents *P* < 0.01 for Δ*toxR*/pBAD33 vs. WT/pBAD33 or C-Δ*toxR*. **(D)** The enterotoxicity was evaluated by determining the fluid accumulation in the ileal loop.

The virulent activity of WT/pBAD33, Δ*toxR*/pBAD33, and Δ*toxR*/pBAD33-*toxR* (C-Δ*toxR*) were performed using the listed phenotypes. First, the survival rates of mice infected with WT, Δ*toxR*, and PBS (negative control) were determined, and the lethality in mice for Δ*toxR* significantly decreased relative to WT and PBS (Figure 3A). We did not use the strains carrying the pBAD33 or pBAD33-*toxR* plasmid because of the lack of arabinose in mice for efficiently inducing expression of pBAD33-*toxR* (Sun et al., 2014). Secondly, cytotoxicity against HeLa cells was investigated regarding the release of LDH from cultured cells (Figure 3B). The cytotoxicity of cells infected with Δ*toxR*/pBAD33 was hugely enhanced compared with that infected with WT/pBAD33 and C-Δ*toxR*. Thirdly, the hemolytic activity was measured by using KP test on the Wagatsuma agar (Figure 3C), and the hemolytic activity of Δ*toxR*/pBAD33 significantly decreased than that of WT/pBAD33 and C-Δ*toxR*. Finally, the enterotoxicity of *V. parahaemolyticus* strains were examined by using a rabbit ileal loop model (Figure 3D), and the results showed that the enterotoxicity of Δ*toxR*/pBAD33 significantly decreased in fluid accumulation compared with the WT/pBAD33 and C-Δ*toxR*. Taken together, ToxR inhibits the cytotoxicity, but it activates mouse lethality, hemolytic activity, and enterotoxicity of *V. parahaemolyticus*.

### Negative regulation of T3SS1 by ToxR

**Figure 4:**
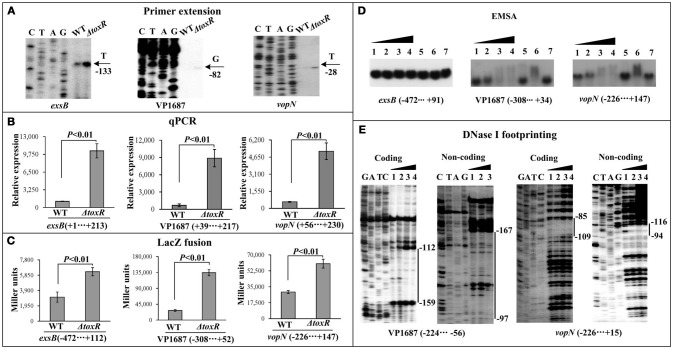
Regulation of T3SS1 genes by ToxR. The primer extension **(A)** and qPCR **(B)** were done as Figure 1, the LacZ fusion **(C)** and EMSA **(D)** assays were done as Supplementary Figure 1, while the DNase I footprinting **(E)** was done as Figure 2.

The first genes of putative operons *exsBAD-vscBCD* (VP1700-1688, T3SS1regulation), VP1687-1686 (T3SS1 effector), VP1667-1656 (T3SS1 apparatus) from T3SS1 locus were selected as the target genes (Table 2), and then subjected to primer extension, qPCR, LacZ fusion, EMSA, and DNase I footprinting assays. The primer extension assay (Figure 4A) detected a single transcriptional start site for each target gene, and the mRNA level of each target was enhanced in Δ*toxR* relative to WT. The qPCR (Figure 4B) further confirmed the mRNA levels of the three genes were enhanced in Δ*toxR* relative to WT. To test the action of ToxR on the promoter activity of the target genes, the recombinant *lacZ* fusion plasmid that contains the indicated regulatory region and a promoterless *lacZ* gene was transferred into Δ*toxR* and WT, respectively (Figure 4C). The results disclosed a significantly increased promoter activity of each of the target genes tested in Δ*toxR* relative to WT. The promoter-proximal DNA regions of the above three genes were radioactively labeled, and subjected to EMSA with the purified His-ToxR (Figure 4D). His-ToxR was unable to bind to the upstream DNA fragment of *exsB*. However, it was able to bind to the upstream DNA fragments of VP1687 and VP1667. As further determined by DNA footprinting (Figure 4E), His-ToxR protected a single DNA region upstream of VP1687 and VP1667, respectively, against DNase I digestion, which were considered as the ToxR-binding sites. Taken together, ToxR indirectly represses the transcription of *exsBAD-vscBCD*, while it negatively regulates the transcription of VP1687-1686 and VP1667-1656 in an indirect manner.

### Positive regulation of Vp-PAI (T3SS2 and *Tdh2*) by ToxR

**Figure 5:**
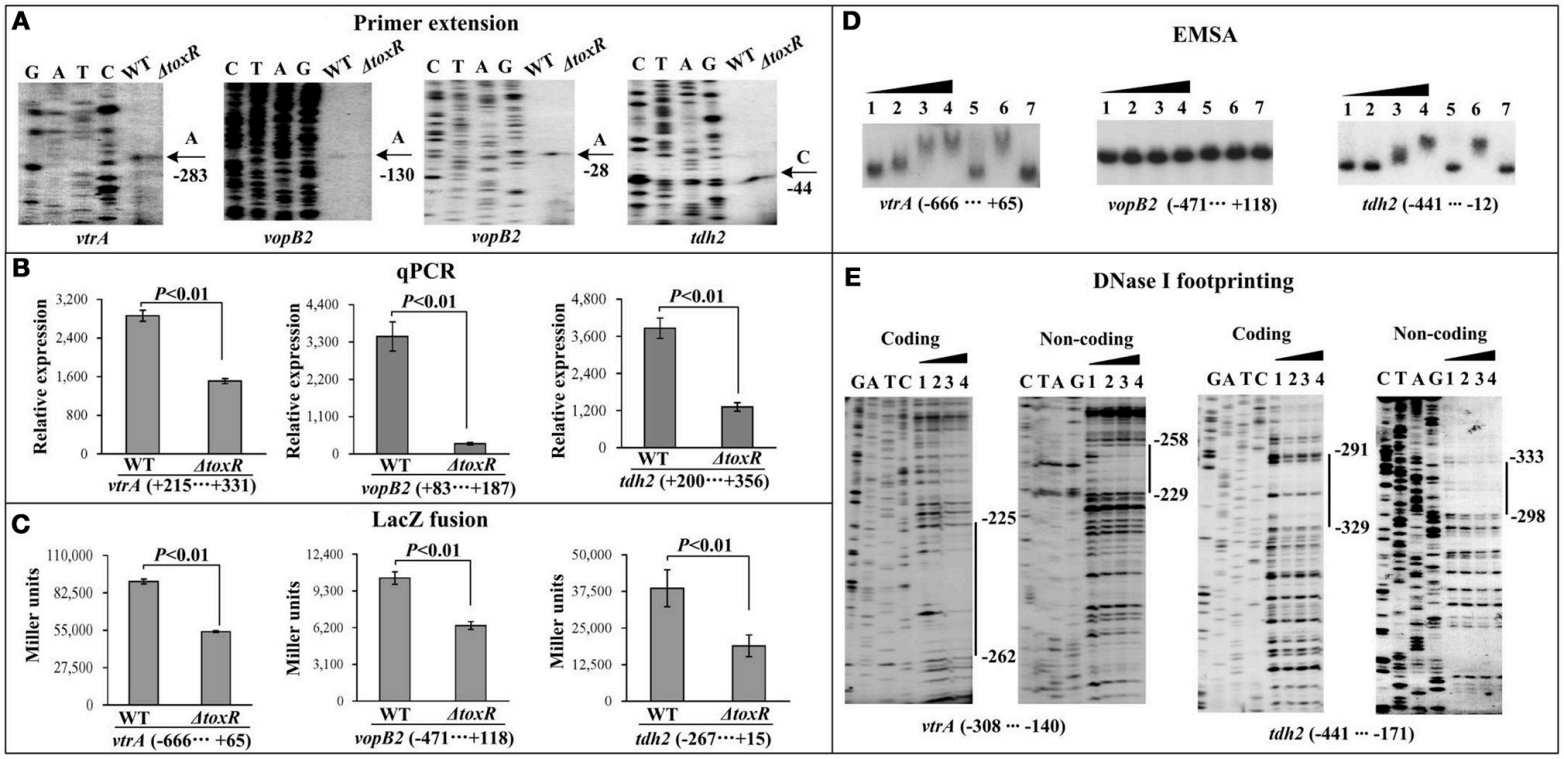
Regulatory actions ToxR on Vp-PAI genes. The primer extension **(A)** and qPCR **(B)** were done as Figure 1, the LacZ fusion **(C)** and EMSA **(D)** assays were done as Supplementary Figure 1, while the DNase I footprinting **(E)** was done as Figure 2.

The first genes of putative operons VPA1332-1333 (Vp-PAI regulation), VPA1362-1358 (T3SS2 apparatus), and *tdh2* from Vp-PAI locus were selected as the target genes (Table 2), and then subjected to the investigation of ToxR-mediated gene regulation via primer extension, qPCR, LacZ fusion, EMSA, and DNase I footprinting assays. The primer extension and qPCR results showed that the transcription levels of all the three genes selected from Vp-PAI decreased in Δ*toxR* relative to WT (Figures 5A,B). The *lacZ* fusion results demonstrated that the promoter activity of each of the three operons in Δ*toxR* was much lower than that in WT (Figure 5C). As further determined by EMSA (Figure 5D), His-ToxR was able to specifically bind to the upstream DNA fragment of *vtrA* (VPA1332) and *tdh2* in a dose-dependent manner, but a negative result was observed for *vopB2* (VPA1362). The results of DNase I footprinting assay showed that His-ToxR protected a single DNA region upstream of *vtrA* and *tdh2* against DNase I digestion, respectively, which were considered as the ToxR-binding sites (Figure 5E). Taken together, ToxR activates the transcription of VPA1332-1333 and *tdh2* in a direct manner, but it indirectly activates the transcription of VPA1362-1358.

## Discussion

A previous study showed that the transcriptional pattern of *toxR* was consistent with that of *aphA*, which was highly expressed at LCD (OD_600_ = 0.05–0.2) when the bacteria were grown in Difco marine broth 2216 (BD Biosciences) at 37°C (Zhang et al., 2017). The phenomenon suggested a possible connection between *toxR* transcription and QS in *V. parahaemolyticus*. In the present work, we showed that the highest transcriptional level of *toxR* occurred at an OD_600_ = 0.2–0.4, but it dramatically decreased when the OD_600_ value was lower than 0.2 or higher than 0.4, suggesting the transcriptional pattern of *toxR* depends on the bacterial growth conditions. We further investigated the regulatory actions of the QS regulators AphA and OpaR on *toxR* transcription. The results showed that AphA indirectly represses the transcription of *toxR* at LCD, whereas OpaR has no regulatory actions on *toxR* transcription. Thus, there should be an additional unknown regulator (s) that contributes to the cell density-dependent transcriptional pattern of *toxR*. We previously showed that CalR occupies the regulatory region of *toxR* to repress its transcription when the bacteria were harvested at the midlogarithmic growth phase (Osei-Adjei et al., 2017), and the expressional level of CalR increased considerably with the increase of cell density from an OD_600_ value of 0.05 to 1.2 (unpublished data). Moreover, we demonstrated herein that ToxR binds to its own promoter-proximal DNA region to repress its own gene transcription at the transition from LCD to HCD (OD_600_ = 0.2–0.4). Thus, the highest transcription of *toxR* occurs at an OD_600_ value of 0.2 to 0.4 and would be due to the subtle regulation of AphA, ToxR, and CalR. Nevertheless, whether there are additional unknown regulators that can regulate the transcription of *toxR* during the growth periods of *V. parahaemolyticus* strain need to be further investigated.

The present work also demonstrated that ToxR acts as an inhibitor of the *V. parahaemolyticus*-induced cytotoxicity against HeLa cells, while it serves as an activator of the lethality in mice, the enterotoxicity in a rabbit ileal model, and the hemolytic activity against RBCs. Also, ToxR binds to the promoter-proximal DNA regions of VP1687-1686 and VP1667-1656 of the T3SS1 locus to repress their transcription, but it manifests indirect repression of *exsBAD-vscBCD* transcription. ToxR, also, occupies the promoter-proximal DNA regions of VPA1332-1333 and *tdh2* of Vp-PAI locus to activate their transcription, but it only has indirect regulatory actions on VPA1362-1358 transcription. Thus, ToxR regulates the multiple virulence phenotypes via directly acting on the T3SS1 and Vp-PAI genes.

T3SS1 is under the subtle regulation of the transcriptional regulatory system ExsACDE in *V. parahaemolyticus* (Zhou et al., 2010; Erwin et al., 2012). ExsA acts as a transcriptional activator of T3SS1 genes (Zhou et al., 2010). ExsD interacts with ExsA to prevent the activation of ExsA (Zhou et al., 2010). ExsC binds ExsD to prevent the binding of ExsD to ExsA, allowing expression of T3SS1 gene (Zhou et al., 2010). ExsE can bind ExsC and, thereby, antagonizes ExsC activity (Erwin et al., 2012). Indirect regulation of *exsBAD-vscBCD* by ToxR indicates ToxR regulation of T3SS1 is not mediated by the ExsACDE system. However, CalR occupies the promoter-proximal DNA region of *exsBAD-vscBCD* to repress its transcription, and ToxR activates *calR*, and CalR feedback inhibits *toxR* and its own gene (Osei-Adjei et al., 2017). Both ToxR and CalR bind to the promoter-proximal DNA regions of one or more operons in T3SS locus to repress their transcription (Osei-Adjei et al., 2017). The operon VPA1332-1333 encodes the transcriptional activator VtrA of Vp-PAI; mutation of *vtrA* leads to reduced fluid accumulation in the rabbit intestine infection model (Kodama et al., 2010). The direct and positive regulatory action of ToxR on the transcription of *vtrA* indicates the entire Vp-PAI genes are under the positive control of ToxR in *V. parahaemolyticus*.

**Figure 6:**
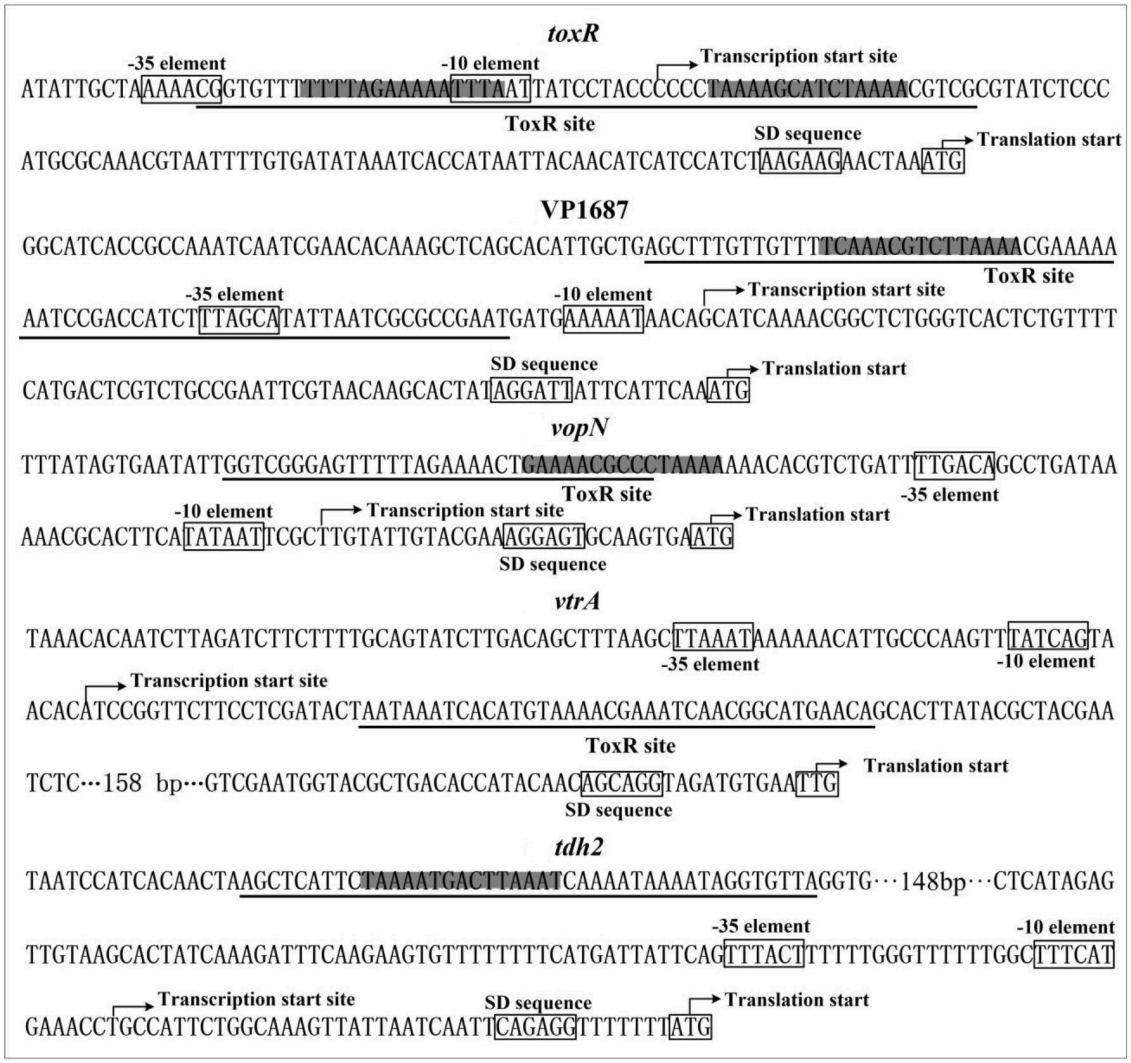
Promoter structure of target genes. The promoter DNA regions of indicated genes were derived from RIMD 2210633. The translation and transcription starts were shown with bent arrows. The predicted core promoter −10 and −35 elements and the SD sequences were boxed. The ToxR box-like sequences were highlighted, and the ToxR sites were underlined.

The organization of *toxR*, VP1687-1686, VP1667-1656, VPA1332-1333, and *tdh2* promoters were reconstructed herein, by collecting the data of translation/transcription start sites, the core promoter −10 and −35 elements, ToxR binding sites, ToxR box-like sequences, ribosomal binding Shine–Dalgarno (SD) sequences (Figure 6). The ToxR-binding sites for both *toxR* and VP1687-1686 overlap the core −35 and/or −10 elements, and thus ToxR is thought to silence the transcription of *toxR* and VP1687 −1686 by directly interfering with RNA polymerase (RNAP) action. The ToxR binding site for VP1667-1656 was detected in the upstream of the −35 element, which is unusual for a regulator that represses its target gene transcription. The binding site of ToxR to *vtrA* promoter is located downstream of the transcription start site, which is also unusual for a regulator that stimulates its target gene transcription. However, similar regulatory mechanisms have been observed in other species, such as *S. enterica* (Shi et al., 2004), *E. coli* (Munson and Scott, 2000), and *Y. pestis* (Zhang et al., 2013). The ToxR-binding site for *tdh2* was located upstream of the promoter −35 element. Thus, the ToxR-stimulated *tdh2* promoter may have a class I transcriptional stimulation that depends on the subunit C-terminal domain of RNAP to function (Ishihama, 2000). It should be noted that not all ToxR-binding sites contain the ToxR box-like sequences, indicating the computational analysis is not a panacea.

Taken together, the present work reports of the autoregulation of ToxR and its regulatory actions on major virulence gene loci in *V. parahaemolyticus*, which is beneficial to the pathogenesis of the pathogen. The data presented here also provide vital information for a deeper understanding of the regulatory patterns of ToxR in *V. parahaemolyticus*.

## Author contributions

DZ, XH, and RY conceived the study and designed experimental procedures. YiqZ, LH, YinZ, WY, ZY, RL, XS performed the experiments and carried out data analysis. YiqZ, GO-A, DZ, and XH wrote the paper.

### Conflict of interest statement

The authors declare that the research was conducted in the absence of any commercial or financial relationships that could be construed as a potential conflict of interest.
