# Supplementary material for: Autoregulation of ToxR and Its Regulatory Actions on Major Virulence Gene Loci in Vibrio parahaemolyticus
Source: Front Cell Infect Microbiol. 2018 Sep 5;8:291. doi: 10.3389/fcimb.2018.00291 (PMC6135047; doi:10.3389/fcimb.2018.00291)
Supplement: Supplementary file 1 [file Data_Sheet_1.docx]

**Supplementary Material**

**
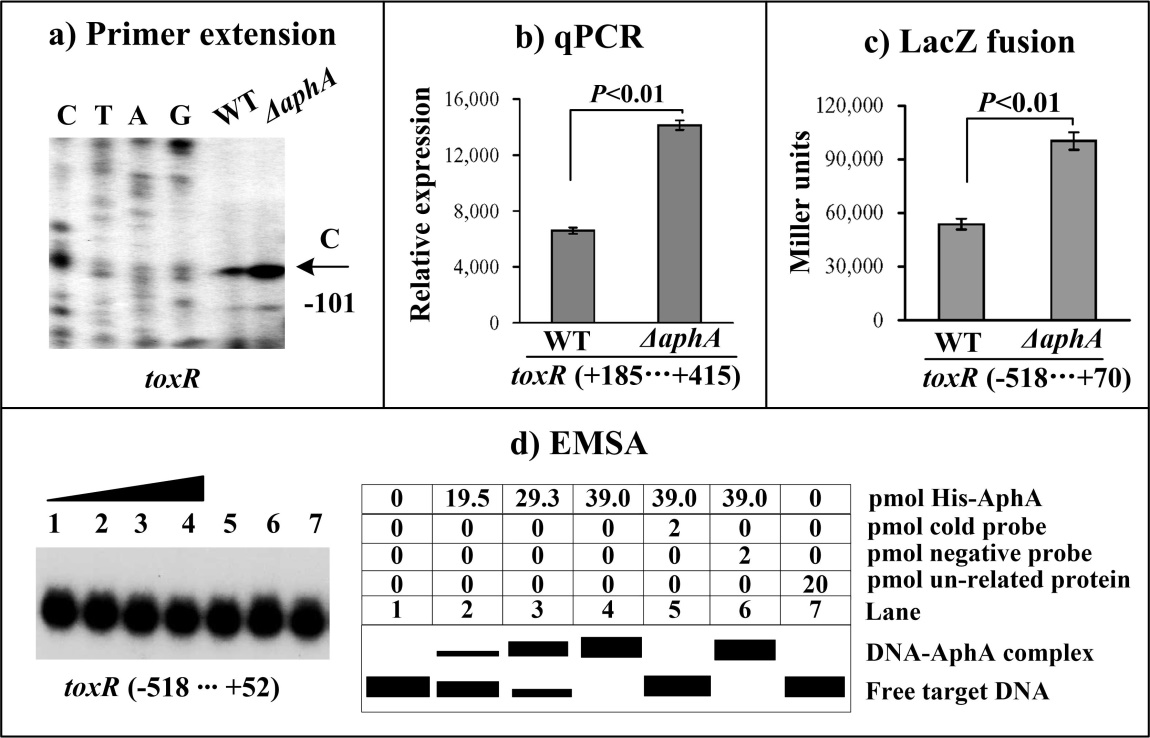
**

**Fig. S1 Regulation of *toxR* by AphA.** Lanes C, T, A, and G represent the Sanger sequencing reactions. The negative and positive numbers indicated the nucleotide positions upstream and downstream of *toxR*. Primer extension (a) and qPCR (b) were done as Fig. 1. **c) LacZ fusion.** The promoter-proximal DNA region of *toxR* was cloned into the *lacZ* transcriptional fusion vector pHRP309 and then transferred into WT and *ΔaphA* to determine the promoter activity (miller units) in the cellular extracts. **d) EMSA.** The radioactively labeled promoter DNA fragments of *toxR* were incubated with increasing amounts of purified His-AphA, and then subjected to 4% (w/v) polyacrylamide gel electrophoresis. Shown also was the schematic representation of the EMSA design.

**
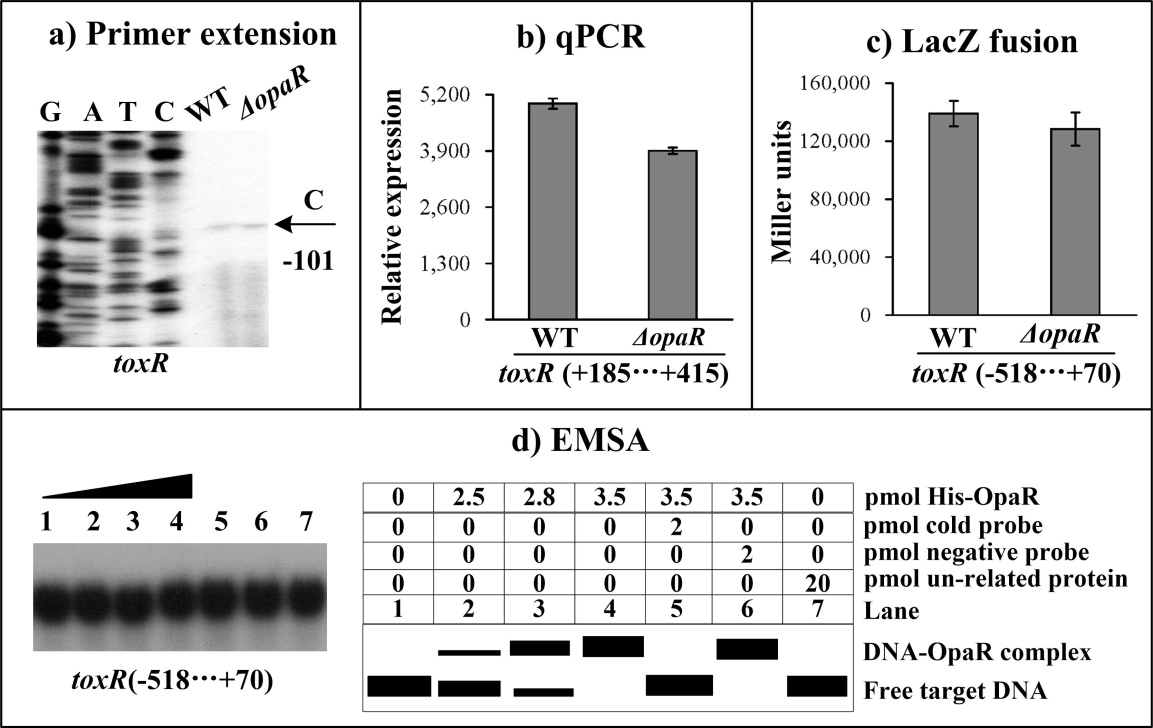
**

**Fig. S2 Regulation of *toxR* by OpaR.** The primer extension (a) and qPCR (b) assays were done as Fig. 1, while the LacZ fusion (c) and EMSA (d) assays were done as Fig. S1.

**
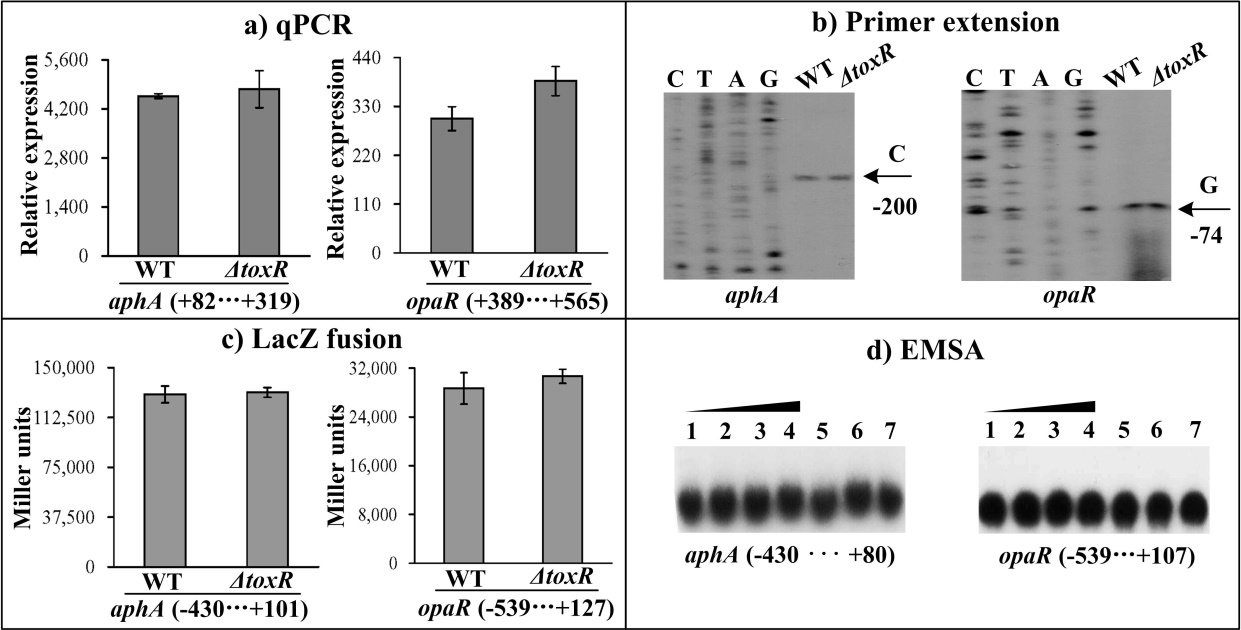
**

**Fig. S3 Regulation of *aphA* and *opaR* by ToxR.** The primer extension (a) and qPCR (b) were done as Fig. 1, while the LacZ fusion (c) and EMSA (d) assays were done as Fig. S1.
